# Classical Olfactory Conditioning in the Oriental Fruit Fly, *Bactrocera dorsalis*

**DOI:** 10.1371/journal.pone.0122155

**Published:** 2015-04-02

**Authors:** Jia Li Liu, Xiao Yan Chen, Xin Nian Zeng

**Affiliations:** Key Laboratory of Natural Pesticide and Chemical Biology of the Ministry of Education, College of Natural Resources and Environment, South China Agricultural University, Guangzhou, Guangdong, China; Tohoku University, JAPAN

## Abstract

The oriental fruit fly, *Bactrocera dorsalis*, is a serious pest of fruits and vegetables. Methyl eugenol (ME), a male attractant, is used to against this fly by mass trapping. Control effect may be influenced by learning, which could modify the olfactory response of the fly to this attractant. To collect the behavioral evidence, studies on the capability of this fly for olfactory learning are necessary. We investigated olfactory learning in male flies with a classical olfactory conditioning procedure using restrained individuals under laboratory conditions. The acquisition of the proboscis extension reflex was used as the criterion for conditioning. A high conditioned response level was found in oriental fruit flies when an odor was presented in paired association with a sucrose reward but not when the odor and sucrose were presented unpaired. We also found that the conditioning performance was influenced by the odor concentration, intertrial interval, and starvation time. A slight sensitization elicited by imbibing sucrose was observed. These results indicate that oriental fruit flies have a high capacity to form an olfactory memory as a result of classical conditioning.

## Introduction

Learning, especially associative learning, tends to adapt an insect to its current environment [1]. Insects learn to orient toward stimuli that are rewarding and away from stimuli that are punishing. Thus, the success of a foraging task strongly depends on learning [2, 3]. The learning processes underlying foraging behavior have been broadly studied in honey bees [4, 5] and bumble bees [6]. Additionally, the recognition of learning as a source of behavioral plasticity in parasitoid foraging has also increased [7, 8].

The oriental fruit fly, *Bactrocera dorsalis* (Hendel), is a serious quarantine pest of fruits and vegetables [9]. Males of *B*. *dorsalis* are strongly attracted to methyl eugenol (ME) [10,11], a naturally occurring compound found in varying amounts in more than 200 species from 32 plant families [12]. A combination of ME with insecticides is being used successfully in “male annihilation” programs against this fly in mass trapping experiments [13–15]. However, *B*. *dorsalis* fed ME are less likely to be captured in ME-baited traps than ME-deprived males [16, 17], and *B*. *dorsalis* avoids ME traps of the same type in which they were caught [18]. We hypothesize that flies may learn to link odor cues and punishment events during feeding or from past experience, which caused the decreased responsiveness to ME. Many phytophagous insects forage for food, hosts, habitats, and mate selectively by learning which is considered to be a searching strategy to locate an appropriate source [19]. In this context, our test flies may also change their olfactory behaviors by learning to make a success searching.

In several species of tephritid fruit flies, the effects of learning on host-finding behavior have been reported. Prior host experience enhances the acceptability of familiar food for the apple maggot fly, *Rhagoletis pomonella* (Walsh) [20, 21], and females of the wild Mediterranean fruit fly, *Ceratitis capitata* (Wiedemann) can learn fruit size to find the host [22, 23]. In *B*. *dorsalis*, a role for olfactory learning in oviposition behavior was demonstrated by Yu [24], who found that female *B*. *dorsalis* laid more eggs on the host that they had experienced as larvae than on novel hosts. However, few studies document olfactory learning in *B*. *dorsalis* adults [25].

To study the olfactory learning capability of this fly, a manageable conditioning procedure is required. We used a classical olfactory conditioning procedure [26–28], which allows control of the sensory experience of a test animal, particularly in regulating its responsiveness to a conditioned stimulus [1]. The acquisition of the proboscis extension reflex (PER) was chosen to evaluate the learning performance. The PER is widely used in classical conditioning to evaluate learning and memory [6, 26, 27], and ME induces this foraging behavior. Thus, we expect that the use of PER will be appropriate for us to apply aversive conditioning in future studies with *B*. *dorsalis*.

In this study, we aimed to investigate the feasibility of using the PER in a bioassay with individual flies to evaluate olfactory learning and to analyze the influence of specific elements on the learning performance of *B*. *dorsalis*.

## Materials and Methods

### Insects

A laboratory strain of *B*. *dorsalis* was established in 2010 in Guangzhou, China. The flies were housed in cages (30 × 30 × 30 cm) and reared on an artificial diet (3:1 sugar to hydrolyzed yeast) and water under laboratory conditions (25–27°C, 60–80% relative humidity, and 14:10 h light: dark photocycle). Banana (*Musa acuminate* Colla) was provided every three days for oviposition. A semiartificial diet that contained banana, yeast, sugar, corn meal, sodium benzoate, fiber and water was used for larval culture, and the mature larvae were transferred to sand for pupation.

The male flies used in this study were obtained from the laboratory colony and were separated from females within 24 h after emergence. For separating, males were gently sucked into a tube (2 cm o.d. × 9 cm long) from the colony and then transferred into a new rearing cage (30 × 30 × 30 cm) with food and water. Eighteen hours before the start of training (except for the groups starved for various periods to investigate the influence of starvation time on olfactory conditioning) a group of 20–30 flies was isolated from the male colony and maintained in a cage (30 × 30 × 30 cm) without food and water to increase the motivation for sucrose. To maintain consistent motivation, during isolation, flies were sucked into a tube whose inner wall was smeared with sucrose solution, and they were allowed to feed freely on sucrose until no feeding behavior was observed. The tube was quite small (2 cm o.d. × 9 cm long), and all flies laid on the wall. Thus, it was easy for flies to feed on sucrose and convenient for observation. Each fly was harnessed in a small, plastic tube (3.5 cm long) with its forelegs and head extending from a cut, narrow end (0.4 cm o.d.) on the tube (S1 Movie). After harnessing, the insects were allowed to acclimatize for 1 h in the experiment room, and were trained between 9:00 and 11:00 h in all experiments. Flies that died during the training were discarded but this mortality was less than 5%. Flies that responded spontaneously to odor before conditioning were excluded, and only those individuals who showed the reflex to sucrose after starvation were considered for the experiments. The flies used in the experiments were 16–18 days old, and each fly was used only once.

### Olfactory conditioning

The classical olfactory conditioning protocol employed in this study was based on the paired association of an odor (conditioned stimulus CS) and a sucrose solution (unconditioned stimulus US), which is commonly applied in honey bees, *Apis mellifera* [26]. Animals that were individually harnessed for training were prepared in squads of 20–30 each. Peppermint oil (Pretty Valley, Guangzhou, China) was used as the conditioned stimulus (CS) and was dissolved in methanol. The odorant concentrations indicated below refer to the volume ratio (v/v) of peppermint oil/ (peppermint oil + solvent). A 10% sucrose solution (DAMAO, Tianjin, China) was used as the appetitive unconditioned stimulus (US). For the conditioning trial, the flies were introduced into the conditioning location individually. After 10 s of adaptation to the airflow, the peppermint odor was delivered for 6 s. Three seconds after the onset of odor presentation, the forelegs of the fly were touched with sucrose to elicit PER, and the sucrose solution was provided for 3 s as a reward. The proboscis extension response to the odor was recorded during the first 3 s.

### Odor stimulus delivery

**Fig 1:**
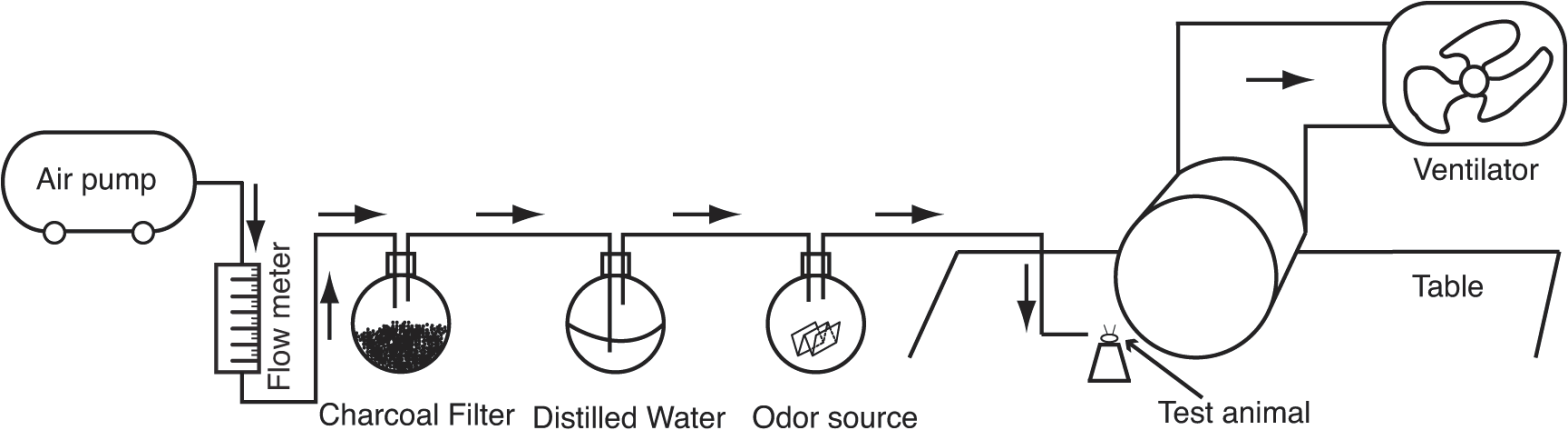
Diagram of the odor delivery system. Arrows indicate direction of the airflow.

A push-pull airflow system was used to provide the odor stimulus (Fig 1). The airflow, supplied from a mini air compressor (FB-36/7, Jiebao, Zhejiang, China), was cleaned with a charcoal filter and remoistened in a bubbler. The flow rate was regulated by a flow meter with a needle valve (LZB-3, Yinhuan, Zhejiang, China). A filter paper (4 × 4 cm) was soaked with 100 μl peppermint solution of a certain concentration, air-dried, and then placed into a glass bottle. After passage through the odorized contents of the bottle, the airflow (20 l/min) was directed to the head of the test flies for 6 s to present the CS. An exhaust fan was placed opposite the stimulus delivery site to immediately extract the odor from the experimental room. The filter paper was changed after each training trial.

### Experimental protocol

#### Paired and unpaired training

During paired training, the flies experienced six conditioning trials with a 10-min intertrial interval (ITI), and the proboscis extension responses to the 20% peppermint solution were recorded in each trial.

During unpaired training, the flies were subjected to twelve trials: six CS-only trials, in which 20% peppermint alone was presented for 6 s, and six US-only trials, in which the sucrose solution was presented for 3 s. The trials were alternated with a 5-min ITI. The CS-only trial was conducted first to remove the animals that responded spontaneously to the odor stimulus. The proboscis extension responses during the first 3 s of the CS-only trials were recorded for analysis.

#### Effect of presenting the CS or US alone

Unlike the unpaired training procedure, in which the CS-only and US-only trials were alternated within the same group, in this experiment, each group was subjected to either CS-only trials or US-only trials. In the CS-only group, the flies were conducted with six CS-only trials with a 10-min ITI, and the proboscis extension responses to 20% peppermint during the first 3 s in each trial were recorded. In the US-only groups, there was no odor, and the US was presented to the flies for 3 s. Five groups of flies were used, and each one was subjected to one, two, three, four, or five US-only trials, respectively. The ITI was set for 10 min. Ten minutes after presentation of the last sucrose US, 20% peppermint was presented for 6 s, and the proboscis extension responses during the first 3 s were recorded. The possible participation of non-associative learning, such as sensitization and habituation, was determined using the responses of the CS- and US-only groups.

#### Effect of odor concentration

The effect of different odor concentrations on the conditioning performance of flies was tested. To four groups of flies were presented 100 μl of the peppermint-methanol solution with concentration of 2%, 10%, 20%, and 40% as the odor stimuli. The flies were trained to associate the odor CS with the sucrose solution US in six conditioning trials with 10-min ITIs. The conditioned proboscis extension response in each trial was recorded for the four groups.

#### Effect of intertrial intervals

To observe the effect of the ITI, five groups of flies were subjected to six conditioning trials with ITIs of 1, 3, 5, 10, and 15 min, and the 20% peppermint solution was paired with the sucrose solution for conditioning. The conditioned proboscis extension response in each trial was recorded for the five groups.

#### Effect of starvation time

We compared the conditioned proboscis extension responses of four groups of flies that were deprived of food and water for various periods. Flies were starved for 1, 12, 18, or 24 h after fed with satiation. The feeding method was introduced above (see ‘Insects‘) except for 1-h group. In this group, after the flies were harnessed in the tubes, they were fed sucrose solution until they did not extend their proboscis, and then they were left for 1 h in the laboratory before the experiment. The remaining three groups of flies were kept in different chambers for starvation. The flies were conditioned to associate 20% peppermint with sucrose for six trials with 10-min ITIs. The conditioned proboscis extension response in each trial was recorded for the four groups.

### Data analyses

#### Conditioned response

The proportion of proboscis extension response in each conditioning trial was recorded. The effect of successive training was analyzed using the Cochran test (*Q*). The proportion of conditioned response between experimental groups in a single test was compared using the G-test for contingency tables. The frequencies of proboscis extension response in the five US-only groups were compared with the responses before training (spontaneous response level) using multiple comparison χ^2^ tests, significance was corrected with the Brunden correction, where α^′^ = α/2(*k*-1), being *k* the number of samples.

#### Learning performance

The number of proboscis extension responses gained along the six odor presentations in the experimental procedure was recorded. Each individual was ranked on the basis of this number (from 0 to 6 positive responses). We used the Kruskal-Wallis (KW) and Scheffé post hoc tests to evaluate the performance levels among more than three groups, and the Mann-Whitney U test to compare the performance levels between two groups [29].

## Results

### Paired and unpaired training

**Fig 2:**
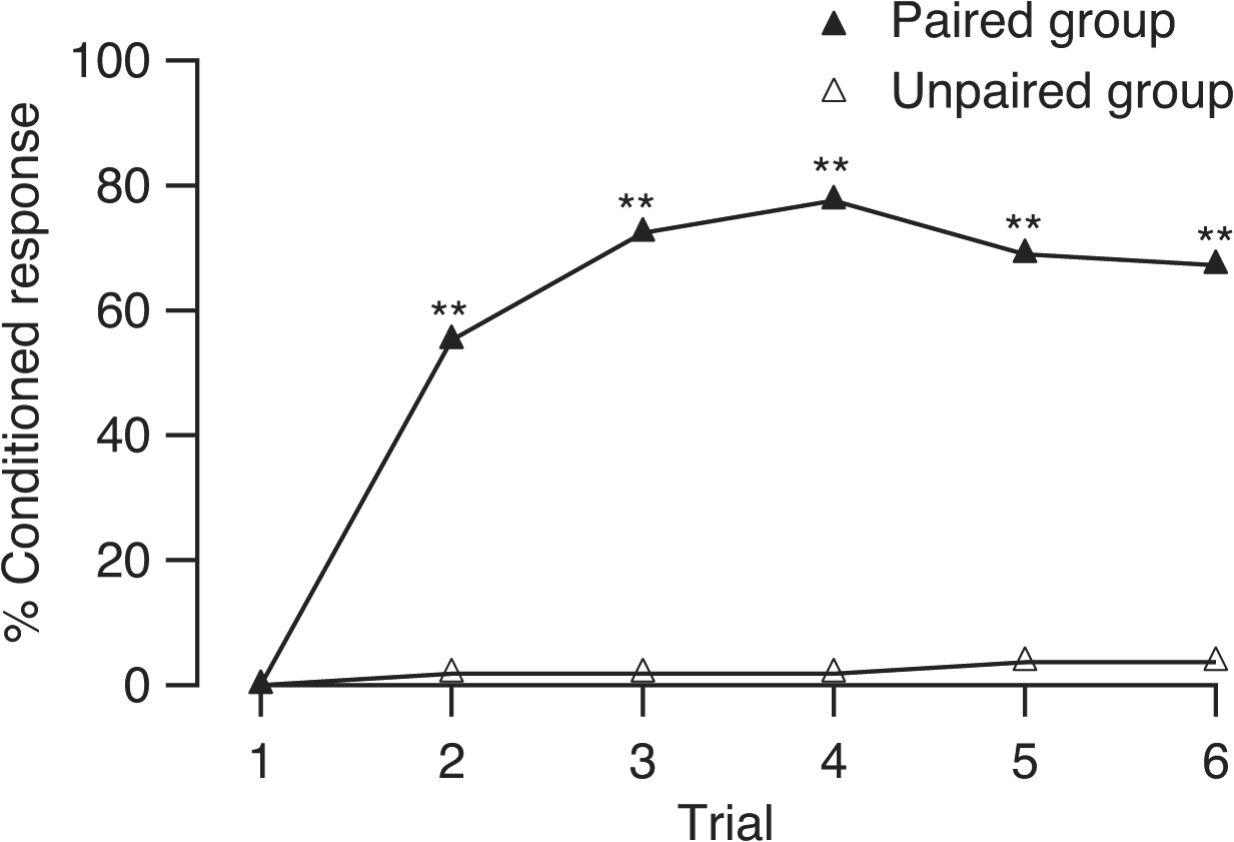
Olfactory conditioning of the proboscis extension response in *B*. *dorsalis*. Percentages of *B*. *dorsalis* showed conditioned response to the CS (% conditioned response) in 6 trials with paired training (*n* = 58) and unpaired training (*n* = 55). The results of statistical comparison paired and unpaired conditioning in each training trial are shown as asterisks (G—test, **p < 0.01)

We first investigated whether the frequency of conditioned response increased during CS presentations with paired training. The first trial was excluded from analysis because it was designed to have a null response, and null response first trial in other experiments also was excluded from analysis. The percentage of conditioned response to the CS significantly increased with the subsequent five successive conditioning trials (Cochran’s test *Q* = 15.77 df = 4, *p* < 0.01; Fig 2) and reached a maximum of 77.6% at the fourth trial (after three training trials). Of the 58 flies, 7 failed to show the conditioned response to the peppermint odor.

By comparing with a control group, the unpaired group, we demonstrated that the increased probability of eliciting a proboscis extension was conditional upon an association between the odor and the sucrose solution. The unpaired group produced a negligible response: one fly responded 4 times, and three others responded once each. The rate of proboscis extension did not increase during training (*Q* = 1.5 df = 4, *p* > 0.5; Fig 2). The performance levels in the two groups were significantly different (Mann-Whitney *U* = 236.5, *p* < 0.001). Differences in frequency of proboscis extension between the paired and unpaired groups were already significant in trial 2, with a higher percentage of responses observed in the paired group than in the unpaired group (*G* = 47.37, df = 1, *p* < 0.01). This difference was also observed over the subsequent training trials (trial 3: *G* = 72.73; trial 4: *G* = 82.01; trial 5: *G* = 60.95; trial 6: *G* = 58.31, all df = 1, *p* < 0.01). These results demonstrate that olfactory memory in the flies was only formed with paired conditioning.

### Effect of presenting CS or US alone

**Fig 3:**
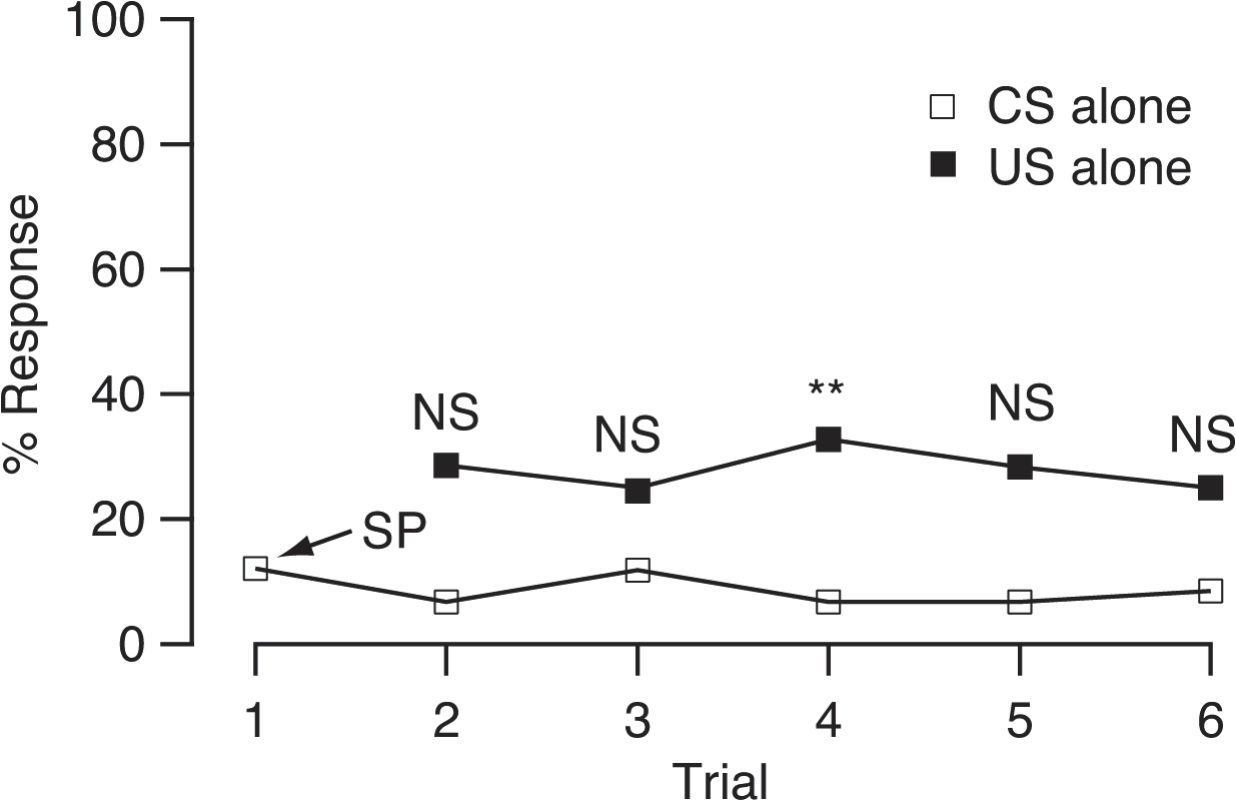
Effects of presenting the CS or US alone. Percentages of *B*. *dorsalis* that extended their proboscis in response to the CS (% response) in a CS-only group (*n* = 59) in 6 trials and in five US-only groups presented with the US alone on one (*n* = 63), two (*n* = 57), three (*n* = 64), four (*n* = 60), and five (*n* = 60) occasions. In the US-only training, proboscis extension responses of five groups were tested 10 min after fed sucrose solution 1–5 times. The trial number was feeding time plus one, such example: the proportion of proboscis extension in the group fed sucrose solution one time is shown at trial 2. The percentage of flies responding to CS before training was the spontaneous response level (SP). Differences between before and after feeding sucrose are denoted with asterisks (*p < 0.05; NS *p* > 0.05)

Because the olfactory and gustatory stimuli used as the CS and US, respectively, might modify the proboscis extension response to the CS by habituation, sensitization or other non-associative learning processes, the training was conducted by presenting only the CS or US alone to the flies. In the CS-only group, the frequency of proboscis extension was 12.1% in trial 1 and this value also refers to a spontaneous response level. After repeating the presentation of peppermint (5x), the percentage of proboscis extension did not increase during training, remaining at 7–12% in trials 1 to 6 (*Q* = 1.33, df = 4, *p* = 0.86; Fig 3). In US-only groups, response frequency was compared before and after fed with sucrose solution. The percentage of proboscis extension before feeding was the spontaneous response to CS. The flies responded significantly more often to peppermint compared with the spontaneous response level after conducting three US-only trials (χ^2^ = 10.0, df = 1, α^′^ = 0.005, *p* < 0.05), and no differences were found in the other groups conducted trials number one, two, four, and five (χ^2^ = 6.98, 4.36, 6.7, and 4.69, respectively, all df = 1, α^′^ = 0.005, *p* > 0.05)

### Effect of odor concentration

**Fig 4:**
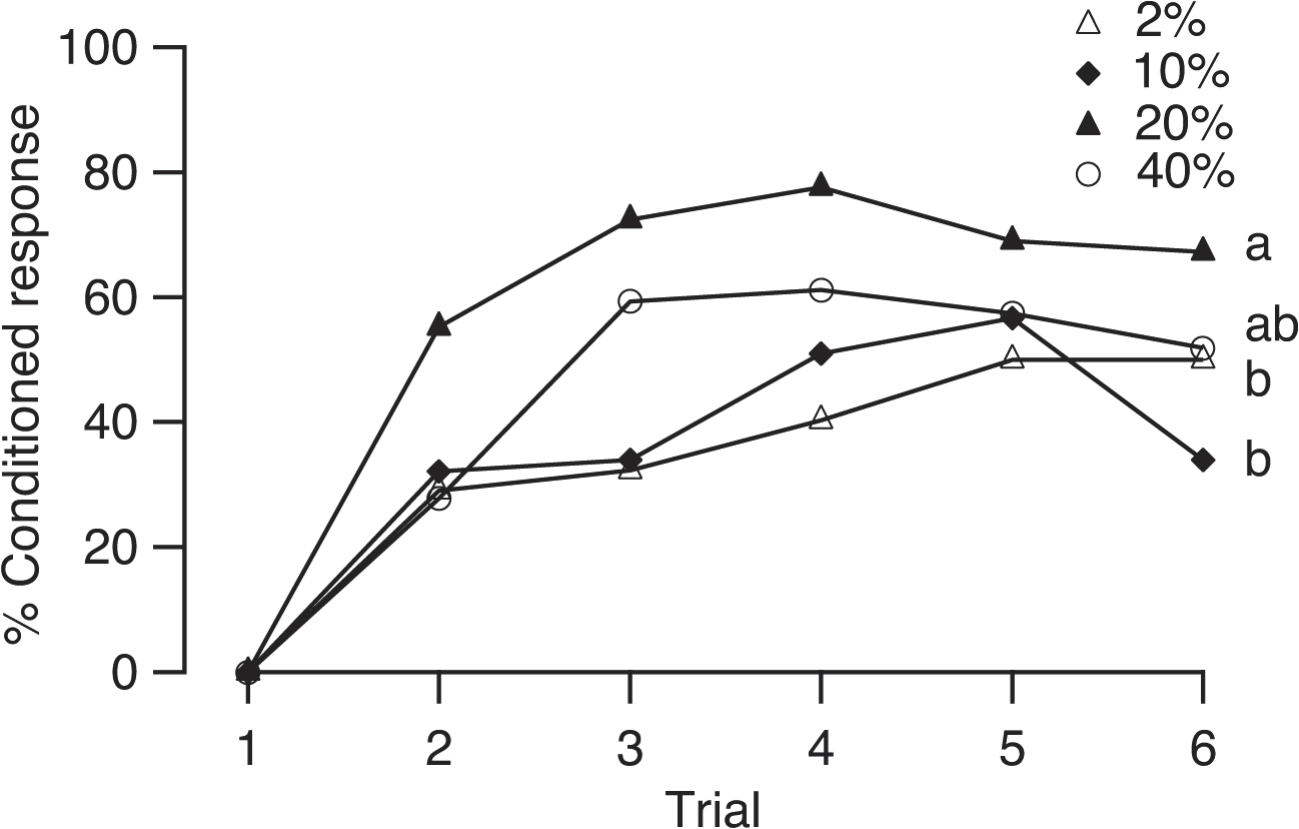
Effects of the odor concentration on conditioning. Percentage of *B*. *dorsalis* showed conditioned response to the CS (% Conditioned response) in 6 conditioning trials at four concentrations: 2% (*n* = 62), 10% (*n* = 53), 20% (*n* = 58), and 40% (*n* = 56). Different letters indicate significant pairwise differences in learning performance (Scheffé test after the Kruskal-Wallis test).

One hundred μl of a peppermint solution in methanol with concentrations at 2%, 10%, 20%, and 40% were presented to the flies for conditioning to test the effect of CS concentration on conditioning. The frequency of conditioned response during five conditioning trials at the four concentrations increased significantly (2%: *Q* = 20.86; 10%: *Q* = 22.58; 20%: *Q* = 16.79; 40%: *Q* = 35.2; all df = 4, *p* < 0.01; Fig 4), reaching a maximum of 50, 56.6, 77.6, and 61.1%, in trial five at 2% and 10% concentrations, trial four at 20% concentration, and trial three at 40% concentration, respectively.

The responses of individual flies over the consecutive trials were affected by the CS concentration. In the 20% group, 88% of flies responded at least once to the CS, compared with only 61.3% in the 2% group, 64.2% in the 10% group, and 68.5% in the 40% group (*G* = 13.52, df = 3, *p* < 0.01). The Kruskal-Wallis test showed that the odor concentration also significantly influenced the performance levels (KW, *H* = 16.47, df = 3, *p* < 0.01) with the levels of performance of groups 2% vs 20% and groups 10% vs 20% being significantly different (Scheffé test, *p* < 0.01 for both comparisons). Although no difference was found between the 20% and 40% groups (Scheffé test, *p* > 0.05), the training effect occurred more rapidly in the 20% group than that in the 40% group after one training session. Difference in response frequency between these two groups in trial 2 was significant (*G* = 8.77, df = 1, *p* < 0.01).

### Effect of intertrial intervals

**Fig 5:**
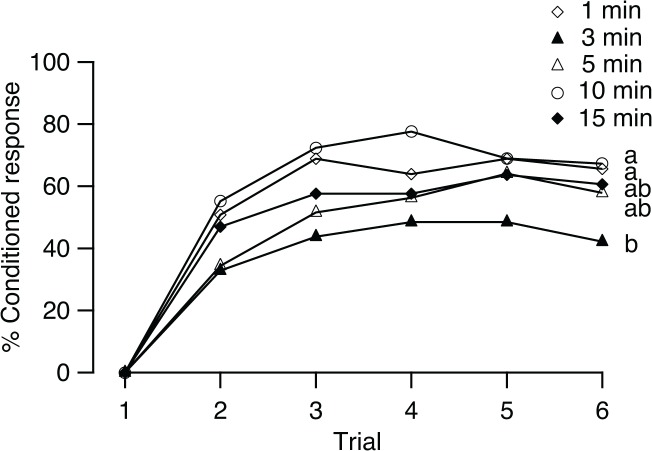
Effects of intertrial intervals on conditioning. Percentage of *B*. *dorsalis* showed conditioned response to the CS (% Conditioned response) in 6 conditioning trials with intertrial intervals of 1, 3, 5, 10, and 15 min (*n* = 59, 64, 64, 58, and 63, respectively). Different letters indicate significant pairwise differences in learning performance (Scheffé test after the Kruskal-Wallis test).

The effect of ITI on conditioning was investigated in the training of five groups of flies in six conditioning trials with ITIs of 1, 3, 5, 10, and 15 min, respectively. In these groups, the percentages of conditioned response increased significantly with successive trials (1 min: *Q* = 10; 3 min: *Q* = 30.02; 5 min: *Q* = 34.8; 10 min: *Q* = 16.79; 15 min: *Q* = 13.7; all df = 4, *p* < 0.05; Fig 5), reaching a maximum of 68.9%, 48.4%, 64.1%, 77.6%, and 63.6%, at the second trial in the 1-min group, the fourth trial in the 3-min group, the fifth trial in the 5-min group, the fourth trial in the 10-min group, and the fifth trial in the 15-min group, respectively.

The ITI affected the responses of individual flies over the consecutive trials. The percentages of flies that responded at least once to the CS during conditioning in these five groups were different (G = 11.34, df = 4, *p* < 0.05). The performance levels were also significantly different among the five groups (KW, *H* = 15.77, df = 4, *p* < 0.01). Scheffé’s post hoc test revealed differences in two comparisons (*p* < 0.05): the 3- vs 1-min groups and the 3- vs 10-min groups.

### Effect of starvation time

**Fig 6:**
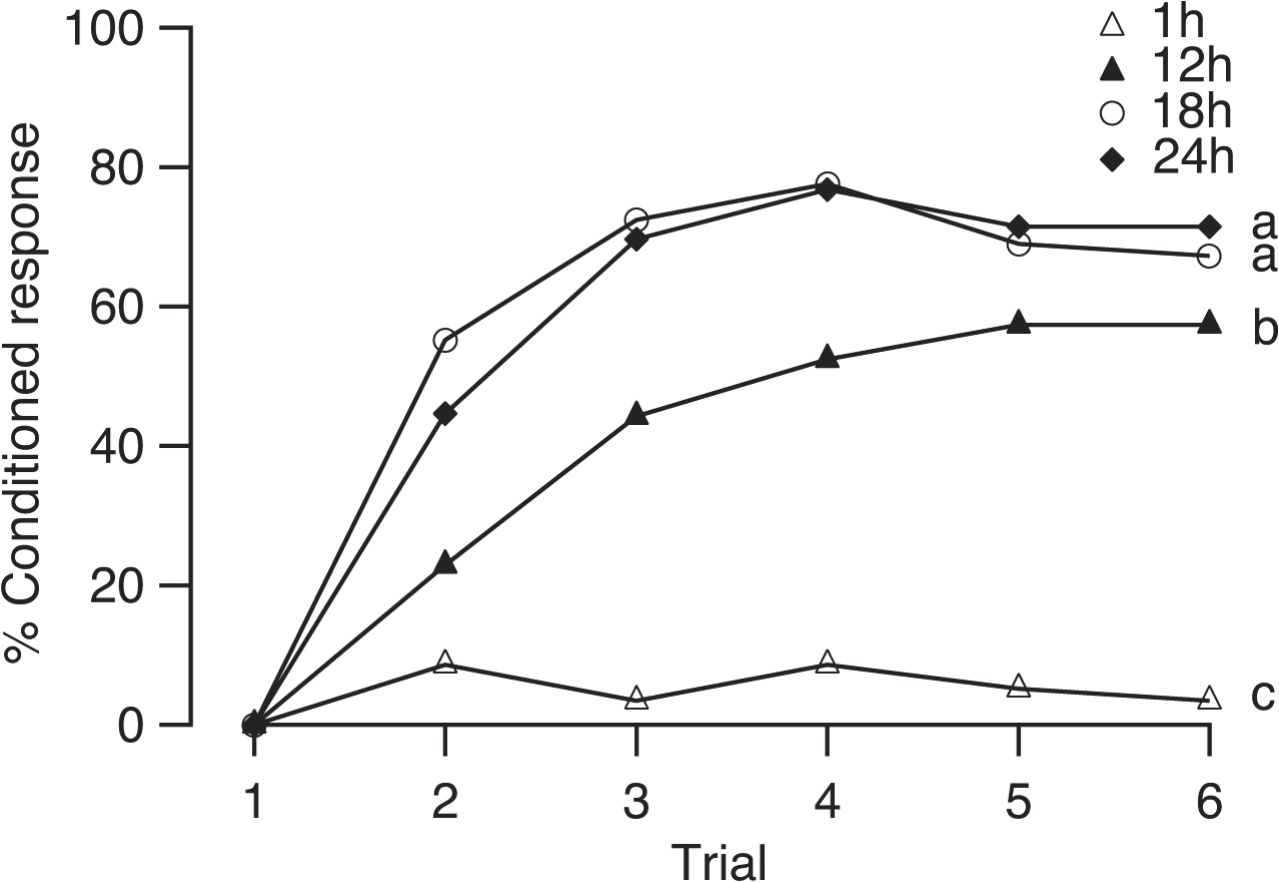
Effects of starvation on conditioning. Percentage of *B*. *dorsalis* starved for 1, 12, 18, and 24 h showed conditioned response (% Conditioned response) to the CS in 6 trials (*n* = 58, 61, 58, and 24, respectively). Different letters indicate significant pairwise differences in learning performance (Scheffé test after the Kruskal-Wallis test).

We assessed whether the PER conditioning was also affected by the starvation time. There was a significant increase in the frequency of conditioned response over the five successive trials after flies were starved for 12, 18, or 24 h (12 h: *Q* = 33.81; 18 h: *Q* = 16.79; 24 h: *Q* = 25.71; all df = 4, *p* < 0.01; Fig 6), and the maximum rate was 57.4, 77.6, and 76.8%, respectively, at the fifth trial in the 12-h group and the fourth trial in the 18-h and 24-h groups. The 1-h group showed response rates lower than 10% in trials 2 through 6 that did not increase significantly during training (*Q* = 7.23, df = 4, *p* = 0.12).

Regarding individual learning performance, the percentages of flies that exhibited at least one conditioned proboscis extension response to the CS in the four groups were significantly different (*G* = 93.88, df = 3, *p* < 0.01). The performance level was significantly influenced by the starvation time (KW, *H* = 85.53, df = 3, *p* < 0.01). The level in the 1-h group was significantly lower than that in the other three groups (Scheffé test for three comparisons, *p* < 0.01). Comparisons of the 12- vs 18-h groups and 12- vs 24-h groups were also different (Scheffé test, *p* < 0.05). No significant difference in performance was found between the 18- and 24-h groups (Scheffé test, *p* > 0.05).

## Discussion

We used a classical olfactory conditioning paradigm to demonstrate that restrained *B*. *dorsalis* can learn to associate a specific odor with a food reward. We succeeded for the first time that PER can serve as a behavioral criterion to monitor the acquisition of association in restrained flies. The comparison between paired and unpaired training experiments showed that a temporal relation between the CS and US is necessary to obtain conditioned responses to the CS. The increased rate of proboscis extension during training was due to the contiguity of CS and US (paired) and not by contextual cues (unpaired). The probable associative basis for the conditioning was similar to those previously observed for bees [6, 26], moths [27], and desert locust [30] although the rates of conditioned responses in these insects were species-specific.

The study of olfactory learning requires appropriate experimental procedures. Using restrained flies with the PER procedure, which is commonly used in conditioning of restrained honey bees [26], bumble bees [6], fruit flies [31], and moths [27], will help to screen out quality/quantity parameters of odor recognition in *B*. *dorsalis* as a good control of the stimulations during training. Our results also proved that the intensity, number, duration and sequence of stimulus, and inter-trial interval had a crucial influence on learning.

The other advantage of this conditioning procedure is that only one specific sensory stimulus was paired with US reward, which is benefit to determine a certain type of learning, such as olfactory learning or visual learning. In a previous study on learning in *B*. *dorsalis* [25], flies were allowed to experience the natural host fruit freely, and they may associate visual or odor cues (or both) with a reward. Thus, the influence of learning on searching for a host might be complex.

The possible involvement of non-associative components in changes of proboscis extension response frequency, such as sensitization or habituation, caused by the materials used as the CS or US, was confirmed with the CS-only and US-only treatments [32, 33]. The response level was not affected by the repeated presentation of the CS odor (Fig 3). However, we observed a higher proportion of proboscis extension response to the CS odor after flies were fed with 10% sucrose solution, a relatively low concentration. Although this trend was found in the five US-only groups, only groups fed with sucrose solution three times showed statistical significance. This may indicate some sensitization during training. Based on this result, caution should be taken in using a high concentration of sucrose solution for conditioning *B*. *dorsalis*. Higher sucrose concentrations may elicit greater sensitization, and an increment of conditioned responses after training may be mainly due to a sensitization process, not to an associative process. This sensitization effect elicited by imbibing sucrose has also been reported in the honey bee [34]. In this case, after a single sucrose stimulus, the probability of a proboscis extension response to an odor stimulus increases more than two-fold and then decreases quickly to the initial level (after approx. 2 min). In the present study, the sensitization effect lasted more than 10 min. Additional experiments need to be conducted to determine the retention time of the sensitization effect and, thus, to know whether the sensitization involves long-term memory.

Stimulus intensity is an important determinant for learning. Our results suggest that high odorant concentration support stronger associations. This finding is consistent with those obtained for honey bees, showing that more intense odors are generally learned faster [35–37]. From the perspective of ecology, a high concentration of odor is more detectible than a lower concentration, making it easier for insects to locate host plants that produce a higher intensity odor [38, 39]. The role of odorant intensity in overshadowing may provide another explanation; that is, during training, insects could also associate a mechanical stimulus (air puff) with a reward, and high concentrations of odorant have a stronger capacity to overshadow the learning of a simultaneously trained mechanical stimulus, inducing a better training efficiency [36].

The behavioral response after conditioning with a certain ITI is related to the memory formation process. Menzel *et al*. found that after a single conditioning trial, there is a U-shaped retention function with a “dip” at 3 min in honey bees, and this biphasic performance is due to a rapidly decaying short-term memory overlying a slower process of the formation of mid-term memory [40]. Learning is especially susceptible to interference during the transformation of these two forms of memory. For 3-mins ITIs, memories established on consecutive trials may be disrupted by each other’s consolidation during that high interference susceptibility [41]. We observed that learning performance was lower after conditioning with an ITI of 3 min than with an ITI of 1 or 10 min. This non-monotonous ITI-dependent learning observed in our studies matched that reported in bees. To avoid the disruption on memory consolidation in mass training trials, it is necessary to study the memory consolidation process after training in detail in our future studies in *B*. *dorsalis*.

Associative learning is also strongly dependent on the motivational status of the subjects. Previous studies in honey bees [34] have shown that intervals after feeding and the amount of feeding influence the rate of the conditioned response. The more bees are starved, the better their training performance is. Our results showed that the intervals after feeding to satiation (starvation time) also affected learning performance in *B*. *dorsalis*. The rate of conditioned response tested for six conditioning trials rose with the increase in time after feeding to satiation. However, we found that the conditioned response level observed at 24 h after feeding to satiation did not increase compared with the level at 18 h. This finding suggests that a ceiling effect of starvation may exist. We also observed that the mortality after 24 h of starvation exceeded 50%, which was significantly higher than the 10% mortality rate observed after 18 h of starvation. Hence, we choose to apply 18 h of starvation in our other experiments because a balance between motivation with hunger and mortality may be necessary.

The successful use of the PER on individual *B*. *dorsalis* flies to evaluate olfactory learning supports its possible use to evaluate the aversive learning with PER in our future research. We found ME can elicit PER, and this ME-elicited PER may decrease when *B*. *dorsalis* link the odor with a negative effect. After confirmed the aversive learning capability, we will attempt to explore whether the preference of flies to ME may also be changed by odor learning.

## Supporting Information

S1 Movie A movie showing how flies were harnessed. A small tube was used to collect the test fly that has been starved for a certain period in a cage. Degreasing cotton was placed in the tube to prevent fly creeping down. Forelegs and head of the harnessed fly can move freely.
(MP4)
